# Negligible Rebound in Myopia Progression Following Cessation of Treatment with 0.01% Atropine for 3 years: Year-4 Results from the CHAMP Phase 3 Clinical Trial

**DOI:** 10.1007/s44402-026-00064-w

**Published:** 2026-04-08

**Authors:** Karla Zadnik, Erica Schulman, Ian Flitcroft, Jennifer S. Fogt, Louis C. Blumenfeld, Tung M. Fong, Eric Lang, Simon P. Chandler, Houman D. Hemmati

**Affiliations:** 1 The Ohio State University College of Optometry, Columbus, Ohio USA; 2 SUNY College of Optometry, New York, New York USA; 3 Centre for Eye Research, Dublin, Ireland; 4 Eye Physicians of Central Florida, Maitland, Florida USA; 5 Vyluma Inc., Bridgewater, New Jersey USA; 6 Present Address: Channel Therapeutics, North Brunswick, New Jersey USA

**Keywords:** Atropine, Myopia control, Randomised controlled trial, Rebound effect

## Abstract

**Introduction:**

Stage 2 of the Childhood Atropine for Myopia Progression (CHAMP) study was conducted to observe the safety, efficacy and rebound effects of low-dose atropine sulphate ophthalmic solutions (0.01% and 0.02%) for paediatric myopia in the year following cessation of 3 years of prior treatment.

**Methods:**

The study was a multicentre, randomised, double-masked, placebo-controlled Phase 3 clinical trial. Children aged 3 to < 17 years with myopia (spherical equivalent refractive error (SER) from −0.50 to −6.00 D) who entered and completed Stage 1 (3 years of treatment) participated in the fourth-year follow-up (Stage 2). Stage 1 participants who were treated with atropine 0.01% or 0.02% were re-randomised 1:1:1 to atropine 0.01%, atropine 0.02% or vehicle in Stage 2. Stage 1 vehicle-treated participants were re-randomised 1:1 to atropine 0.01% or atropine 0.02% in Stage 2. Outcomes included changes in cycloplegic SER, changes in axial length (AL), responder status continuity and progression to high myopia.

**Results:**

Four hundred and twenty participants were randomised in Stage 2. Negligible rebound was observed among the participants who switched from 0.01% atropine to vehicle compared to those who continued on 0.01% atropine treatment (least square mean difference in SER change from Month 36 to Month 48 between continued treatment and switching to vehicle = 0.019 D, 95% CI [−0.14, 0.18], *p* = 0.82 for the modified Intent to Treat set). Stage 1 responders remained responders in 83-90% of participants in various Stage 2 groups. Safety profiles were favourable, with mild, transient adverse events.

**Conclusions:**

Atropine sulphate ophthalmic solution (0.01%) exhibited negligible rebound in myopia progression after treatment cessation following 3 years of continued treatment, highlighting its potential as a treatment option. The treatment maintained a favourable safety record, and the overall benefit and risk assessment support its potential use as a long-term myopia management option.

**Clinical trial registration:**

The Childhood Atropine for Myopia Progression (CHAMP) study was registered in clinicaltrials.gov (NCT03350620, registration date November 17, 2017).

Key Points

- The current randomised, double-masked study demonstrated negligible rebound in myopia progression following cessation of 3 years of treatment with 0.01% atropine, which slowed myopia progression in children.
- For participants who previously achieved stable myopia over 3 years with continuous treatment using 0.01% atropine, treatment cessation may be considered in clinical practice.
- For children aged 3–16 years, treatment of 0.01% atropine for up to 4 consecutive years was well tolerated. No new safety signal was identified.

## Introduction

Juvenile-onset myopia is a growing global concern, with increasing prevalence and the potential for progression to high myopia, which is associated with significant ocular pathologies and visual impairment, including sight-threatening complications such as retinal detachment, glaucoma and myopic maculopathy [1–4]. Effective interventions to slow myopia progression are critical in reducing the risk of complications later in life [5].

Atropine eye drops have emerged as an effective pharmacological intervention for myopia control [6]. Early studies, such as the atropine for the treatment of childhood myopia (ATOM) and the low-concentration atropine for myopia progression (LAMP) studies, established the efficacy of high-to-medium concentrations (1–0.05%) of atropine but revealed significant adverse effects, including photophobia, accommodation deficits and notable rebound effects after treatment cessation [7, 8]. In the ATOM trial, rebound was defined as faster myopia progression after stopping atropine treatment compared with those who never received atropine or pre-cessation [7]. Subsequent trials demonstrated that lower concentrations (0.05–0.01%) balanced efficacy with tolerability, but also reported a dose-dependent rebound proportional to the concentration of atropine [8–13]. The LAMP study defined rebound as faster myopia progression in those who stopped receiving atropine eye drops compared with those who stayed on continued treatment [13].

The Childhood Atropine for Myopia Progression (CHAMP) Phase 3 trial evaluated NVK002, a novel preservative-free, room-temperature stable, atropine sulphate formulation in a single-use container. CHAMP was a multicentre, randomised, double-masked, placebo-controlled study designed to evaluate the safety and efficacy of two concentrations of atropine sulphate ophthalmic solution, 0.01% and 0.02%, over a 3-year period (Stage 1). The results of Stage 1 demonstrated significant reductions in myopia progression for 0.01%-treated participants in all pre-specified secondary endpoints with minimal adverse effects, while 0.02% was significant in some pre-specified secondary endpoints (such as axial length (AL) and time-to-event endpoints) but not significant for the responder and the SER change from baseline endpoints [14, 15]. The CHAMP study results, along with the findings from other studies using 0.01% atropine, suggested that this concentration is a safe and effective option for long-term myopia management [8–10, 12, 14, 16–23]. Of the eight published randomised placebo-controlled studies of 0.01% atropine with a treatment duration of 2 years, seven found evidence of superiority for 0.01% vs. placebo [10, 14, 17, 18, 20–22], whereas one study did not find evidence of superiority for 0.01% atropine vs placebo [24].

A key aspect of myopia management, particularly with pharmacological intervention, is understanding the potential for rebound in myopia progression upon treatment cessation or dose reduction. Understanding rebound is critical, as it affects decisions regarding the duration of treatment and strategies for discontinuing therapy. While the CHAMP study’s Stage 1 focused on establishing the 3-year efficacy and safety of low-dose atropine in controlling myopic progression, extension into a 4th year (Stage 2) offered an opportunity to observe changes in ophthalmic parameters upon dose reduction or treatment cessation, and the potential rebound in myopia progression following treatment cessation. The CHAMP study’s Stage 2 evaluated the rebound in progression, similar to the LAMP study washout phase, except that CHAMP Stage 2 compared continued atropine treatment versus switching from atropine to vehicle treatment with a double-masked design [14], whereas the LAMP washout phase compared continued treatment versus no treatment in an unmasked design [13].

Stage 2 of the CHAMP study in the fourth year sought to address two questions: (1) do these lower doses of atropine mitigate the potential of rebound seen with higher doses of atropine and (2) what is the safety profile with extended use of low-dose atropine? These inquiries are crucial for delineating the long-term utility of atropine in the clinical management of juvenile-onset myopia, guiding clinicians in optimising treatment duration and developing protocols for tapering or discontinuing treatment based on individual patient responses. This report describes findings from Stage 2 of the CHAMP study.

## Methods

The CHAMP Phase 3 trial, sponsored by Vyluma Inc. (vyluma.com), was designed as a two-stage randomised, multicentre, double-masked, placebo-controlled study across the United States and Europe (20 sites in the US and 6 sites in Europe, including the UK, Ireland, Hungary, the Netherlands and Spain) [14]. The study assessed the efficacy and safety of atropine sulphate ophthalmic solution in two concentrations (0.01% and 0.02%) for controlling juvenile-onset myopia progression. The study protocol was approved by appropriate regulatory authorities and applicable institutional review boards or ethics committees, and the study was conducted following principles of Good Clinical Practice and in compliance with the Declaration of Helsinki. The CHAMP study was registered in clinicaltrials.gov (NCT03350620), where the study protocol and statistical analysis plan can be found.

Eligible participants for Stage 1 included children aged 3–<17 years [14]. The primary objective of the study in Stage 1 was to evaluate myopia progression over 3 years in children aged 6–10 years at baseline [14]. The inclusion of children aged 3–5 years and 11–<17 years was to comply with the Paediatric Research Equity Act [25]. Stage 2 was a continuation with an exploratory objective to observe the safety and efficacy in participants re-randomised to 1 year of treatment with atropine sulphate ophthalmic solution, 0.01% or 0.02% or vehicle following 3 years of treatment in Stage 1. Participants were required to have a spherical equivalent refraction (SER) of −0.50 to −6.00 D at the Stage 1 screening and astigmatism not >1.50 D in either eye. Eligible participants for Stage 2 included children who had successfully completed Stage 1 without significant adverse events or protocol violations. Participants who discontinued treatment in Stage 1 or discontinued the study were not eligible. The inclusion and exclusion criteria for participants in CHAMP Stage 1 have been described previously [14]. Written informed consent was obtained from the participants’ legal guardians, and assent was obtained as appropriate. The study enrolled participants with a broad geographical distribution to ensure a diverse participant pool.

Following completion of Stage 1 and for those who re-consented to continue to Stage 2, participants who were treated with atropine 0.01% or 0.02% in Stage 1 were re-randomised into one of the three treatment arms: NVK002 atropine sulphate ophthalmic solution 0.01%, 0.02% or vehicle (placebo). This atropine-to-same/other concentration/vehicle re-randomisation assessed maintenance of myopia control with changes in atropine concentration as well as the rebound effect. Participants assigned to the vehicle group in Stage 1 were re-randomised to one of the two active treatment groups for Stage 2. This vehicle-to-atropine re-randomisation was implemented for ethical reasons. The re-randomisation process was stratified by Stage 1 baseline age and SER, ensuring these characteristics were balanced across the treatment groups. Re-randomisation minimises the potential selection bias that only children who completed Stage 1 were eligible to enter Stage 2. Study medication was administered as one drop in each eye nightly.

Participants were assessed every 3 months for safety during Stage 2, with detailed efficacy and safety evaluations conducted at 6-month intervals. Participants underwent cycloplegic autorefraction and AL measurements every 6 months, with crystalline lens thickness as an optional assessment. Cycloplegic agents (two doses given 5 min apart) were 0.5% or 1% cyclopentolate (for subjects <6 years at randomisation and continuing throughout the study) or 1% tropicamide (for subjects ≥6 years at randomisation and continuing throughout the study). Cycloplegic refraction was conducted with the autorefractor available at the site (either Grand Seiko WR-5100K or WAM-5500 (grandseiko.com), Nidek Tonoref II or OPD Scan III (nidek-intl.com), Tomey Auto Refractor RC 4000 DUAL (tomeyusa.com) or Topcon Auto Kera-Refractometer KR-8900 (topconhealthcare.com)) and the same autorefractor at each site was used throughout the entire study except in cases of instrument malfunction. AL was measured using either the Carl Zeiss Meditec IOLMaster (zeiss.com), Haag Streit LENSTAR (haag-streit.com), Nidek Optical Biometer AL-Scan (nidek-intl.com) or Alladin Biometer (topconhealthcare.com). The same AL instrument was used throughout the entire study at each site, except in the case of instrument malfunction. All SER autorefractor measurements for analysis were converted to the corneal plane (termed normalised SER). A refractive prescription was provided if the refraction revealed at least 0.50 D myopic progression and/or if the eye care practitioner deemed it clinically necessary to improve vision. Additional details of ophthalmic examinations are specified in the study protocol (NCT03350620 in clinicaltrials.gov). Safety evaluations, including adverse event monitoring, were comprehensive and occurred at every quarterly clinic visit. Additional safety assessments, such as heart rate, visual acuity, slit-lamp examination and tonometry, were performed biannually, while dilated fundus examination was performed annually.

### Statistical Analysis

Statistical analysis for CHAMP Stage 2 was designed to assess the rebound potential of atropine sulphate ophthalmic solutions. All efficacy endpoints in Stage 2 were either tertiary or exploratory endpoints. Both the modified intent-to-treat (mITT) and intent-to-treat (ITT) datasets were analysed in Stage 2. The mITT dataset was pre-specified as the main analysis, and the ITT for supportive analyses. The mITT set comprised all participants randomised in Stage 2 who were 6–10 years of age at the initial randomisation into Stage 1, capturing a demographic critically relevant to the study’s objectives on juvenile-onset myopia control. The mITT set was chosen due to its clinical relevance, representing a cohort with a consistent exposure duration and developmental phase, likely to exhibit a uniform response to myopia progression treatment. This selection criterion ensured that the statistical analyses would yield results that are both scientifically robust and clinically meaningful, particularly for paediatric patients at a critical stage of ocular development. The ITT dataset included all participants aged 3–16 years at Stage 1 randomisation. The safety set included participants who had been treated with at least one dose of the study medication in Stage 2, and was the analysis set for safety data summary.

Efficacy endpoint analyses:

- *Tertiary efficacy endpoints*: (1) The change in SER from Stage 2 baseline to Month 48 was analysed using a mixed-effects model for repeated measures (MMRM). The independent variables included treatment group, visit, eye (right or left), baseline age group, baseline SER group and a treatment-by-visit interaction term. Random intercepts for subject and eye-within-subject were included. (2) Rebound assessment based on responders (<0.50 D myopia progression) at Month 42 and Month 48 for participants on active treatment who were responders at Month 36. The responder definition is for analytical purposes and is not a clinical response definition. Summary statistics are provided without modelling.
- Exploratory efficacy endpoints: (1) change from Stage 2 baseline in AL; (2) responder analysis based on <0.75 D myopia progression in SER in Stages 2 and (3) time to change in myopia progression of 0.75 D.

#### Multiplicity

No adjustments were made for multiplicity for the tertiary and exploratory efficacy endpoints analysed in Stage 2.

#### Handling of Missing Data and Statistical Power Considerations

The mixed effects model analysis assumed a Missing at Random missingness mechanism, without imputation of missing data. The statistical power calculation was not performed for Stage 2, as all endpoints in Stage 2 were tertiary or exploratory.

#### Descriptive Statistics

When both eyes are summarised, the median, minimum and maximum are listed in the tables. When subject-level descriptive statistics are summarised, the mean, minimum and maximum are listed in the tables.

#### Statistical Software and Analysis Plan

All statistical analyses were pre-specified in a comprehensive statistical analysis plan and conducted using SAS version 9.4 (SAS Institute Inc., sas.com).

## Results

### Participants and Disposition

**Fig. 1:**
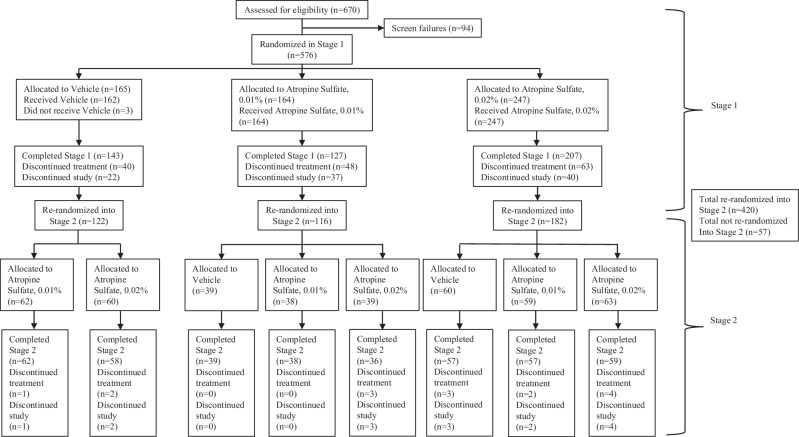
Disposition of participants (ITT set). Details of Stage 1 disposition were reported previously [14]. ITT intent-to-treat.

Study participants’ demographics and Stage 1 baseline characteristics have been described previously [14]. The disposition is summarised in Fig. 1. A total of 420 participants were re-consented and re-randomised in Stage 2. Distribution of participants in each analysis set is summarised in Supplemental Table 1. Demographic and Stage 2 baseline characteristics are summarised in Supplemental Table 2.

### Safety Profile

Safety assessments conducted during Stage 2 revealed that atropine sulphate ophthalmic solutions at both concentrations (0.01% and 0.02%) were well tolerated during the 4th year of treatment. Among the 420 participants who were re-randomised, 174 (41.4%) experienced at least one treatment-emergent adverse event (TEAE). The proportion of participants with TEAEs in the 0.01%, 0.02% and vehicle groups were 47.2%, 39.5% and 35.4%, respectively (Table 1). Ocular TEAEs were infrequent, with the most common being dry eye, occurring in two participants (1.3%) in the 0.01% group and three participants (1.9%) in the 0.02% group (Supplemental Table 3). Other ocular events were reported in ≤1% of participants overall (Supplemental Table 3). Most ocular TEAE’s were not treatment-related as determined by the investigator. No participants in any group experienced a serious ocular TEAE.

**Table 1:** Overall summary of treatment-emergent adverse events—Stage 2 (Stage 2 Safety set).

AE type	Vehicle *N* = 99 *n* (%)	Atropine sulphate 0.01% *N* = 159 *n* (%)	Atropine sulphate 0.02% *N* = 162 *n* (%)
Participants with any TEAE^a^	35 (35.4)	75 (47.2)	64 (39.5)
Participants with any ocular TEAE	5 (5.1)	16 (10.1)	13 (8.0)
Participants with any non-ocular TEAE	32 (32.3)	66 (41.5)	54 (33.3)
Participants with any treatment-related^b^ TEAE	1 (1.0)	2 (1.3)	2 (1.2)
Participants with any ocular treatment-related TEAE	0	2 (1.3)	2 (1.2)
Participants with any non-ocular treatment-related TEAE	1 (1.0)	0	0
Participants with any serious TEAE	2 (2.0)	3 (1.9)	2 (1.2)
Participants with any ocular serious TEAEs	0	0	0
Participants with any non-ocular serious TEAEs	2 (2.0)	3 (1.9)	2 (1.2)
Participants with any treatment-related^b^ serious TEAE	0	0	0
Participants with any TEAE leading to permanent discontinuation of study drug	1 (1.0)	0	1 (0.6)
Participants with any ocular TEAE leading to permanent discontinuation of study drug	0	0	1 (0.6)
Participants with any non-ocular TEAE leading to permanent discontinuation of study drug	1 (1.0)	0	0
Participants with any TEAE leading to death	0	0	0

*AE* adverse event, *TEAE* treatment-emergent adverse event

^a^Adverse events are considered treatment emergent if they began or worsened in severity between the first dose date in Stage 2 and the last dose date of Stage 2.

^b^Adverse events are considered treatment-related if study drug causality is very likely/certain, probable, possible or missing.

Non-ocular TEAEs were reported in 152 participants (36.2%) overall, occurring in 41.5%, 33.3% and 32.3% of the participants in the 0.01%, 0.02% and vehicle groups, respectively (Table 1). The most frequent non-ocular TEAE was COVID-19 infection, reported in 8.0–8.8% of participants across groups (Supplemental Table 3). Most non-ocular TEAE’s were not treatment-related as determined by the investigator. Non-ocular serious adverse events (SAEs) were rare (1.9%, 1.2% and 2.0% in the 0.01%, 0.02% and vehicle groups, respectively) and unrelated to the study medication. No clinically significant changes in heart rate, intraocular pressure, best corrected visual acuity, slit-lamp examination or dilated fundus examination were observed.

### Efficacy Outcomes

#### Spherical Equivalent Refraction (SER)

One of the tertiary efficacy endpoints, the change in SER from Stage 2 baseline to Month 48, evaluated rebound potential when active treatment was changed to vehicle. Throughout this report, various treatment groups were identified by the naming convention of “concentration in Stage 1-to-concentration in Stage 2”. For the mITT set, participants in the 0.01%-to-0.01% group experienced a median change from Stage 2 baseline of −0.17 D (minimum −0.76, maximum 0.24; *n* = 54 eyes), while those in the 0.01%-to-vehicle group exhibited a median change of −0.21 D (minimum −0.75, maximum 0.78; *n* = 52 eyes). The 0.02%-to-0.02% group exhibited a median change of −0.16 D (minimum −1.31, maximum 0.66; *n* = 102 eyes), while those in the 0.02%-to-vehicle group exhibited a median change of −0.19 D (minimum −0.94, maximum 0.94; *n* = 96 eyes). Other mITT treatment groups are summarised in Supplemental Table 4. Similar descriptive statistics were found for the ITT set (Supplemental Table 5). Negligible rebound effects were observed in the mITT set following mixed effects model analysis (Table 2).

**Table 2:** Efficacy assessments in Stage 2 (mITT set).

	Atropine sulphate 0.01% to vehicle *N* = 31	Atropine sulphate 0.01–0.01% *N* = 29	Atropine sulphate 0.02% to vehicle *N* = 52	Atropine sulphate 0.02–0.02% *N* = 56
*SER*				
LS mean change from Stage 2 baseline in normalised SER at Month 48, D	−0.214	−0.195	−0.216	−0.207
95% CI	−0.331, −0.096	−0.309, −0.080	−0.301, −0.130	−0.290, −0.125
LS mean difference (Continuing on same concentration – Switching to vehicle), D	0.019		0.008	
95% CI	−0.144, 0.182		−0.109, 0.125	
*p*-value	0.82		0.89	
*AL*				
LS mean change from Stage 2 baseline in AL at Month 48, mm	0.143	0.116	0.156	0.154
95% CI	0.106, 0.180	0.078, 0.154	0.127, 0.185	0.127, 0.182
LS mean difference (continuing on same concentration–Switching to vehicle), mm	−0.027		−0.002	
95% CI	−0.079, 0.025		−0.041, 0.038	
*p*- value	0.30		0.94	

Mixed effects model was used to analyse the SER and AL endpoints.

*AL* axial length, *D* dioptre, *LS* least square, *mITT* modified intent-to-treat, *SER* spherical equivalent refractive error.

#### Responder Analysis

A further tertiary efficacy endpoint was the rebound assessment of the Stage 1 responders in Stage 2. A responder at eye level in Stage 1 was defined as <0.50 D myopia progression over 3 years (i.e., achieving stable myopia over 3 years). Only those atropine-treated eyes that were responders in Stage 1 are summarised. A responder at eye level in Stage 2 was defined as <0.50 D myopia progression in the 4th year. Overall, the majority of the Stage 1 responders at eye level met the Stage 2 responder criteria for Stage 2 at Month 48, regardless of whether the subject continued on the same dose or transitioned to a lower dose of atropine or vehicle (Table 3).

**Table 3:** Rebound assessment of Stage 1 responder eyes in Stage 2 (Stage 2 mITT set).

Treatment group, number of participants	Number of Stage 1 responder eyes^a^ *n*	Stage 2 responder status^b^	Number and proportion of responder eyes^b^ at Month 42 *n* (%)	Number and proportion of responder eyes^b^ at Month 48 *n* (%)
Atropine sulphate 0.01% to 0.01% *N* = 29	20	Yes	19^c^ (100.0)	18 (90.0)
		No	0^c^ (0.0)	2 (10.0)
Atropine sulphate 0.01% to Vehicle *N* = 31	20	Yes	18^c^ (100.0)	15^c^ (83.3)
		No	0^c^ (0.0)	3^c^ (16.7)
Atropine sulphate 0.02–0.01% *N* = 52	23	Yes	19^c^ (95.0)	16^c^ (88.9)
		No	1^c^ (5.0)	2^c^ (11.1)
Atropine sulphate 0.02–0.02% *N* = 56	26	Yes	21^c^ (87.5)	22 (84.6)
		No	3^c^ (12.5)	4 (15.4)
Atropine sulphate 0.02% to Vehicle *N* = 52	25	Yes	23 (92.0)	22 (88.0)
		No	2 (8.0)	3 (12.0)

*N *= number of participants in Stage 2, *n *= number of eyes.

^a^Stage 1 responder eye is defined as an eye that showed <0.5 D SER progression at Month 36 from the study baseline.

^b^Stage 2 responder eye is defined as an eye that showed <0.5 D SER progression at Month 42 or 48 from the Stage 2 baseline (Month 36).

^c^Total eye number (Yes + No) is different from Stage 1 responder eye number in column 2 due to missing data.

*D* dioptre, *mITT* modified intent-to-treat, *SER* spherical equivalent refractive error.

#### Axial Length

The change in AL aligned with the SER findings. For the mITT set, the 0.01%-to-0.01% group exhibited a median AL change from Stage 2 baseline of 0.10 mm (minimum −0.12, maximum 0.54), while the 0.01%-to-vehicle group exhibited a median AL change from Stage 2 baseline of 0.11 mm (minimum −0.06, maximum 0.45). The 0.02%-to-0.02% group recorded a median AL change from Stage 2 baseline of 0.14 mm (minimum −0.08, maximum 0.55), while the 0.02%-to-vehicle group exhibited a median AL change from Stage 2 baseline of 0.13 mm (minimum −0.20, maximum 0.69). Other mITT treatment groups are summarised in Supplemental Table 4. Similar results were found for the ITT set (Supplemental Table 5). Differences between the continued atropine group and the atropine-to-vehicle group were associated with *p* ≥ 0.30 in the mITT set following mixed effects model analysis (Table 2).

#### Progression to High Myopia

In the mITT population, the proportion of participants who progressed to high myopia (SER ≤ –6.00 D in one or both eyes) ranged from 3.8% to 16.7% across the treatment groups. The study duration of Stage 2 was not long enough to allow a large number of participants to progress to –6.00 D (i.e., Stage 2 median baseline SER = –3.53 D, 75th percentile Stage 2 baseline SER = –4.60 D). Transitioning to vehicle in Stage 2 did not lead to a significantly greater proportion of participants progressing to high myopia compared with the groups of participants who continued on the same concentration (Supplemental Table 6).

#### Clinical and Economic Impact

In the mITT population, approximately half of the participants per treatment group did not receive a new spectacle and/or contact lens prescription during Stage 2 (Table 4). Transitioning to vehicle in Stage 2 did not lead to a significantly greater number of new spectacle and/or contact lens prescriptions compared to groups who continued on the same concentration of atropine in Stage 2.

**Table 4:** Summary of new spectacle and/or contact lens prescriptions given during Stage 2 (mITT set).

	Vehicle to atropine sulphate 0.01% *N* = 54	Vehicle to Atropine sulphate 0.02% *N* = 54	Atropine sulphate 0.01% to vehicle *N* = 31	Atropine sulphate 0.01–0.01% *N* = 29	Atropine sulphate 0.01–0.02% *N* = 33	Atropine sulphate 0.02–0.01% *N* = 52	Atropine sulphate 0.02% to vehicle *N* = 52	Atropine sulphate 0.02–0.02% *N* = 56
Number of new spectacle and/or contact lens prescriptions given due to progression	Number of participants, *n* (%)							
0	25 (47.2)	30 (55.5)	14 (45.2)	16 (55.2)	18 (56.3)	24 (46.2)	21 (42.0)	26 (47.3)
1	20 (37.7)	19 (35.2)	15 (48.4)	9 (31.0)	9 (28.1)	20 (38.5)	22 (44.0)	21 (38.2)
2	8 (15.1)	5 (9.3)	2 (6.4)	4 (13.8)	5 (15.6)	8 (5.3)	7 (14.0)	8 (14.5)
	**Comparison of continuing same atropine concentration vs. switched to vehicle**							
Absolute risk difference of 0 prescription (continuing vs. switched to vehicle)	–	–	10.0%		–	–	5.3%	
95% Wald CI	–	–	−35%, 15%		–	–	−24%, 14%	
*p*-value (continuing vs. switched to vehicle)	–	–	0.44		–	–	0.59	

*CI* confidence interval, *mITT* modified intent-to-treat.

#### Other Efficacy Outcomes

A responder analysis based on less than 0.75 D myopia progression in SER from Stage 2 baseline also indicated negligible rebound for those who switched from atropine in Stage 1 to vehicle in Stage 2, compared with the continued treatment group (Supplemental Table 7). Due to the 1-year duration of Stage 2 and the relatively small change in mean SER during the fourth year (Table 2), there were insufficient participants with an SER change of 0.75 D to allow a meaningful time-to-event analysis of time-to-change in myopia progression of 0.75 D in Stage 2. Crystalline lens thickness changes were similar across all groups (Supplemental Table 8). Pupil size measurements showed an acute pharmacodynamic response to atropine in the vehicle-to-atropine groups (Supplemental Table 9).

## Discussion

The CHAMP Phase 3 clinical trial, encompassing both Stage 1 and Stage 2, represents the first global 4-year study on the long-term safety and efficacy of low-dose atropine sulphate ophthalmic solution for juvenile-onset myopia control. Stage 2 of CHAMP provides key insights into the risk of rebound in myopia progression following treatment cessation or modification and the potential clinical impacts of long-term therapy.

### Negligible Rebound Following Treatment Cessation

**Fig. 2:**
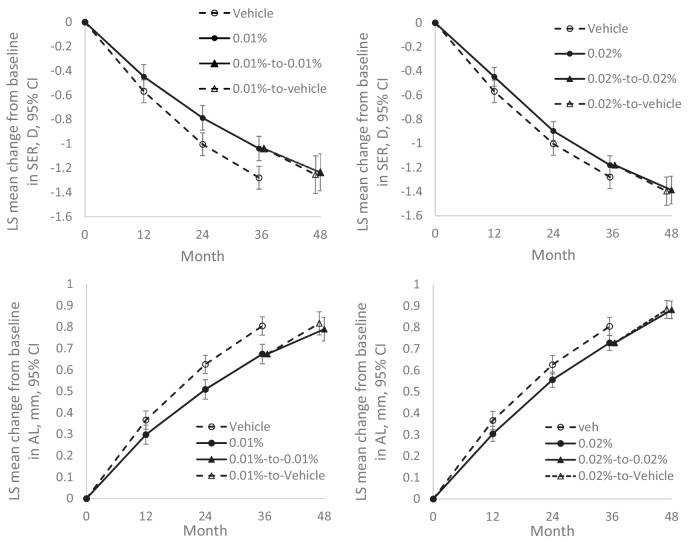
Overall SER or AL progression curves in both Stage 1 and Stage 2 of the Childhood Atropine for Myopia Progression (CHAMP) study over 4 years (mITT set). Least square (LS) mean change from baseline (Month 0) in SER is shown along with 95% CI. Month 0 to Month 36 results [14] from Stage 1 are combined with results from Stage 2 (Table 2). (Top Left) SER change from study baseline for 0.01% in Stage 1, along with 0.01%-to-0.01% or 0.01%-to-vehicle in Stage 2. (Top Right) SER change from study baseline for 0.02% in Stage 1, along with 0.02%-to-0.02% or 0.02%-to-vehicle in Stage 2. (Bottom Left) AL change from study baseline for 0.01% in Stage 1, along with 0.01%-to-0.01% or 0.01%-to-vehicle in Stage 2. (Bottom Right) AL change from study baseline for 0.02% in Stage 1 along with 0.02%-to-0.02% or 0.02%-to-vehicle in Stage 2. AL axial length, D dioptre, LS least square, mITT modified intent-to-treat, SER spherical equivalent refractive error.

The results of Stage 2 support the efficacy of 0.01% atropine in slowing myopia progression. During the 4th year of treatment, the difference among various groups in the changes in SER or AL was negligible, including those transitioning from active treatment to vehicle. The negligible rebound effect is contrasted with the clear treatment effect in Stage 1 (least-square mean difference [0.01%−vehicle] in SER change from baseline over 3 years = 0.24 D [95% CI: 0.106, 0.374]) (Fig. 2) [14]. This finding is particularly important in the context of prior studies, such as the ATOM and LAMP trials [7, 13], which reported notable rebound effects following cessation of higher concentrations of atropine (0.05–1%). The negligible rebound following cessation of 0.01% atropine treatment observed in this study highlights the unique pharmacological profile of 0.01% atropine, making it well-suited for long-term myopia management. The current results are consistent with two other studies of 0.01% atropine in which treatment cessation or a tapered reduction regimen did not lead to significant rebound in myopia progression [11, 26]. The LAMP study reported marginal rebound in SER but insignificant rebound in AL for continued 0.01% treatment versus washout [13]. The Western Australia - Atropine for the Treatment of Myopia (WA-ATOM) study examining cessation of 0.01% atropine did suggest a rebound effect [27], although the observed rebound can be explained by the slowing of progression in the placebo washout group, with no change in the progression trajectory in the 0.01% washout group. The difference in SER or AL trajectories during the washout period between the 0.01% and the placebo washout groups in the WA-ATOM study appears to be confounded by the significantly older age of the placebo group versus the 0.01% group [27] (i.e., an age-dependent decrease in myopia progression rate [28, 29]).

### Responder Continuity and Real-World Implications

An encouraging finding from Stage 2 was the high proportion of Stage 1 responders who maintained responder status in Stage 2, regardless of treatment transition. A majority (83%) of treated eyes transitioning from 0.01% to vehicle remained as responders (i.e., stable myopia), with <0.50 D of myopia progression over the 4th year. This continuity suggests that the benefits of atropine treatment for responders persist even after dose reduction or cessation, an important consideration for clinicians planning long-term treatment courses.

The responder continuity is not only clinically significant but also has real-world implications for patients and families. Stable myopia translates to fewer spectacle prescription changes, reducing the economic burden associated with frequent vision deterioration in children. Approximately half of all participants in Stage 2 did not require optical correction updates, which reinforces the value of low-dose atropine in myopia management. It should be pointed out that the high responder proportion and low optical prescription changes in Stage 2 are likely a reflection of the relatively small SER changes in Stage 2.

### Progression to High Myopia

The progression to high myopia (defined as SER ≤ −6.00 D) was evaluated in CHAMP [14], although the number of participants who progressed to –6.00 D was relatively small (Supplemental Table 6). Longitudinal natural history studies of juvenile-onset myopia indicate the need for enroling younger participants over a much longer follow-up period to observe the progression to high myopia [30, 31].

### Safety and Tolerability

The safety profile observed in Stage 2 reaffirms the tolerability of NVK002 low-dose atropine for use over 4 years. The incidence of ocular and non-ocular TEAEs was low; most were unrelated to study treatment and were mild. Importantly, no serious ocular adverse events were related to the study medication and there were no clinically significant changes in intraocular pressure or visual acuity.

The favourable tolerability of low-dose atropine is contrasted with that of higher concentrations evaluated in earlier studies, which were associated with photophobia, accommodation deficits and rebound effects [7, 8, 32]. The data from CHAMP suggest that the novel preservative-free formulation of 0.01% atropine sulphate ophthalmic solution achieves a safety-efficacy balance, addressing a critical unmet need in paediatric eye care.

Recently, the Stage 2 results of the three-year Myopia Outcome Study of Atropine in Children (MOSAIC) were published [26]. While the Stage 2 design of MOSAIC is different from that of CHAMP, the safety and tolerability profile of 0.01% atropine was consistent across the CHAMP and MOSAIC studies.

### Clinical Implications and Future Directions

The findings from Stage 2 provide a foundation for clinical decision-making regarding the use of 0.01% atropine in myopia management. The treatment effects shown in Stage 1 over 3 years, coupled with the clinically negligible rebound upon cessation or dose reduction, suggest that 0.01% atropine can be integrated effectively into long-term myopia management plans. These results also support flexible dosing strategies, allowing clinicians to tailor treatment duration and concentration to individual patient's needs without compromising efficacy. While the CHAMP trial focused on 0.01% and 0.02% atropine, future studies may explore the integration of atropine treatment with optical interventions, such as multifocal contact lenses, orthokeratology or spectacles to enhance myopia control further.

### Limitations

Despite its robust design, the CHAMP trial has limitations as described previously for Stage 1 [14]. Stage 2’s limitations include the relatively small number of participants per group, efficacy outcomes were exploratory in nature, the risk of type II error and a lack of continuing vehicle control group, which was not possible due to ethical concerns. However, the main purpose of Stage 2 was to observe potential rebound. Additionally, while the study supports sustained efficacy and safety over 3 years in Stage 1 and negligible rebound upon treatment cessation in Stage 2, longer follow-up periods would be desirable to document longer-term implications of treatment and cessation related to myopia control.

## Conclusions

The CHAMP Phase 3 clinical trial, including its fourth-year Stage 2, suggests 0.01% atropine as a safe and effective therapy for juvenile-onset myopia management. By halting or slowing myopia progression while offering a favourable safety profile, this study supported many other published studies on the efficacy of 0.01% atropine, particularly for young children who are not suitable for contact lenses or at high risk of myopia progression. The negligible rebound effect upon cessation and the real-world benefits, such as fewer spectacle and/or contact lens prescription changes, highlight the potential to increase available treatment options in clinical practice and improve outcomes for children with myopia.

## Supplementary information

Supplementary Information

## Data Availability

The datasets are available for collaborative research from Vyluma Inc.
