# Supplementary material for: Negligible Rebound in Myopia Progression Following Cessation of Treatment with 0.01% Atropine for 3 years: Year-4 Results from the CHAMP Phase 3 Clinical Trial
Source: Ophthalmic Physiol Opt. 2026 Apr 8;46(3):626–34. doi: 10.1007/s44402-026-00064-w (PMC13369339; doi:10.1007/s44402-026-00064-w)
Supplement: Supplementary file 1 — Supplementary Information [file 44402_2026_64_MOESM1_ESM.docx]

**Supplementary Information (SI)**

Supplementary content can be found online in the Supplementary Information section.

Table of Contents:

List of additional members of CHAMP Trial Group Investigators

Supplemental Table 1: Subject distribution in each analysis set

Supplemental Table 2: Demographic and baseline characteristics (Stage 2)

Supplemental Table 3: Complete list of treatment-emergent adverse events (Stage 2 safety set)

Supplemental Table 4: Normalised SER or AL change from Stage 2 baseline at Month 48 (Stage 2 mITT set).

Supplemental Table 5: Normalised SER or AL change from Stage 2 baseline at Month 48 (Stage 2 ITT set)

Supplemental Table 6: Proportion of Participants that Progressed to High Myopia (Normalised SER ≤ - 6.00 D) by Month 48 (Stage 2 mITT set)

Supplemental Table 7: Summary of proportion of participants’ eyes that show less than 0.75 D myopia progression from Stage 2 baseline (Stage 2 mITT set).

Supplemental Table 8: Summary of crystalline lens thickness measurements (Stage 2 ITT set).

Supplemental Table 9: Summary of pupil size measurements (Stage 2 ITT set).

**List of additional members of the CHAMP Trial Group Investigators**

| **First Name and Middle Initial(s)** | **Last Name** | Institution | Location (city, state/province, country) |
| --- | --- | --- | --- |
| Carol | Aune | GaGarner Eyecare Center | Raleigh, North Caroline, USA |
| Isabel | Ayet | Hospital Sant Joan de Déu | Barcelona, Spain |
| Darren J. | Bell | Medical Center Ophthalmology Associates | San Antonio, Texas, USA |
| Marie | Bodack | Southern College of Optometry | Memphis, Tennessee, USA |
| Jeffrey | Colburn | Spokane Eye Clinical Research | Spokane, Washington, USA |
| Susan | Cotter | Southern College of Optometry at Marshall B Ketchum University | Fullerton, California, USA |
| Annegret | Dahlmann-Noor | Moorfields Eye Hospital | London, UK |
| Stephen | Glaser | Kids Eye Care of Maryland | Rockville, Maryland, USA |
| James | Hoekel | St. Louis Children's Hospital | St. Louis, Missouri, USA |
| Danielle | Iacono | SUNY College of Optometry | New York, New York, USA |
| Erin | Jenewein | Pennsylvania College of Optometry at Salus University | Philadelphia, Pennsylvania, USA |
| Caroline | Klaver | Erasmus MC | Rotterdam, Netherlands |
| Stephen | Lichtenstein | Illinois Eye Center | Peoria, Illinois, USA |
| Chunming | Liu | Pacific University College of Optometry | Forest Grove, Oregon, USA |
| James | Loughman | Centre for Eye Research Ireland | Dublin, Ireland |
| Zoltan | Nagy | Budapest Retina Intezet | Budapest, Hungary |
| Matthew | Paul | Danbury Eye Physicians | Danbury, Connecticut, USA |
| Melanie | Schmitt | University of Wisconsin-Madison | Madison, Wisconsin, USA |
| David | Silbert | Conestoga Eye | Lancaster, Pennsylvania, USA |
| Sarah | Singh | University of California, Berkeley School of Optometry | Berkeley, California, USA |
| J. Daniel | Twelker | University of Arizona Department of Ophthalmology and Vision Science | Tucson, Arizona, USA |
| Fuensanta | Vera-Diaz | New England College of Optometry | Boston, Massachusetts, USA |
| Dorothy | Wang | Westview Optometry | San Diego, California, USA |
| Colin | Willoughby | Ulster University | Northern Ireland, UK |

Supplemental Table 1: Subject distribution in each analysis set.

|  | Atropine Sulphate 0.01% to Vehicle N=39 n (%) | Atropine Sulphate 0.02% to Vehicle N=60 n (%) | Vehicle to Atropine Sulphate 0.01% N=62 n (%) | Vehicle to Atropine Sulphate 0.02% N=60 n (%) | Atropine Sulphate 0.01% to 0.01% N=38 n (%) | Atropine Sulphate 0.01% to 0.02% N=39 n (%) | Atropine Sulphate 0.02% to 0.01% N=59 n (%) | Atropine Sulphate 0.02% to 0.02% N=63 n (%) | Total  N=420  (%) |
| --- | --- | --- | --- | --- | --- | --- | --- | --- | --- |
| Enrolled in Stage 2 |  |  |  |  |  |  |  |  | 420 |
| Re-randomised for Stage 2 | 39 (100) | 60 (100) | 62 (100) | 60 (100) | 38 (100) | 39 (100) | 59 (100) | 63(100) | 420 (100) |
| Safety set | 39 (100) | 60 (100) | 62 (100) | 60 (100) | 38 (100) | 39 (100) | 59 (100) | 63 (100) | 420 (100) |
| Intent To Treat (ITT) set | 39 (100) | 60 (100) | 62 (100) | 60 (100) | 38 (100) | 39 (100) | 59 (100) | 63 (100) | 420 (100) |
| Modified Intent To Treat (mITT) set | 31 (79.5) | 52 (86.7) | 54 (87.1) | 54 (90.0) | 29 (76.3) | 33 (84.6) | 52 (88.1) | 56 (88.9) | 361 (86.0) |

ITT, intent-to-treat. mITT modified intent-to-treat.

Supplemental Table 2: Demographic and baseline characteristics (Stage 2 safety set).

|  | Atropine Sulphate 0.01% to Vehicle N=39 n (%) | Atropine Sulphate 0.02% to Vehicle N=60 n (%) | Vehicle to Atropine Sulphate 0.01% N=62 n (%) | Vehicle to Atropine Sulphate 0.02% N=60 n (%) | Atropine Sulphate 0.01% to 0.01% N=38 n (%) | Atropine Sulphate 0.01% to 0.02% N=39 n (%) | Atropine Sulphate 0.02% to 0.01% N=59 n (%) | Atropine Sulphate 0.02% to 0.02% N=63 n (%) | Total  N=420  (%) |
| --- | --- | --- | --- | --- | --- | --- | --- | --- | --- |
| Age at Stage 1 baseline | | | | | | | | | |
| Mean (years) | 8.7 | 8.9 | 9.0 | 8.7 | 9.2 | 9.1 | 8.9 | 8.9 | 8.9 |
| Min, Max (years) | 3, 13 | 3, 15 | 5, 15 | 4, 13 | 4, 14 | 5, 14 | 3, 14 | 5, 14 | 3, 15 |
| Age at Stage 2 baseline | | | | | | | | | |
| Mean (years) | 11.7 | 11.9 | 12.0 | 11.7 | 12.2 | 12.1 | 11.9 | 11.9 | 11.9 |
| Min, Max (years) | 6, 16 | 6, 18 | 8, 18 | 7, 16 | 7, 17 | 8, 17 | 6, 17 | 8, 17 | 6, 18 |
| Sex, n (%) | | | | | | | | | |
| Female | 24 (61.5) | 35 (58.3) | 36 (58.1) | 35 (58.3) | 17 (44.7) | 18 (46.2) | 30 (50.8) | 36 (57.1) | 231 (55.0) |
| Male | 15 (38.5) | 25 (41.7) | 26 (41.9) | 25 (41.7) | 21 (55.3) | 21 (53.8) | 29 (49.2) | 27 (42.9) | 189 (45.0) |
| Normalised SER at Stage 2 baseline, combined left and right eyes | | | | | | | | | |
| n | 78 | 120 | 124 | 118 | 75 | 76 | 118 | 126 | 835 |
| Median, D | -3.345 | -3.325 | -3.730 | -3.620 | -3.240 | -3.475 | -3.430 | -3.720 | -3.500 |
| Min, Max | -7.45, -0.66 | -7.80, 0.05 | -7.78, -0.68 | -7.88, -0.80 | -7.69, -0.80 | -7.03, -0.39 | -7.78, -0.61 | -7.05, -0.25 | -7.88, 0.05 |
| Axial Length at Stage 2 baseline, combined left and right eyes | | | | | | | | | |
| n | 78 | 120 | 124 | 120 | 74 | 78 | 118 | 126 | 838 |
| Median, mm | 24.910 | 24.640 | 25.355 | 24.970 | 25.190 | 24.900 | 25.075 | 25.000 | 24.990 |
| Min, Max | 23.14, 27.09 | 22.73, 26.85 | 22.88, 27.42 | 23.05, 27.17 | 23.29, 27.13 | 23.43, 27.61 | 22.92, 28.40 | 22.90, 27.70 | 22.73, 28.40 |

D, dioptre. SER, spherical equivalent refractive error. Min, minimum. Max, maximum.

**Supplemental Table 3: Complete list of treatment-emergent adverse events (Stage 2 safety set)**

| System Organ Class  Preferred Term | Vehicle N=99 n(%) | Atropine Sulphate 0.01% N=159 n(%) | Atropine Sulphate 0.02% N=162 n(%) | Total N=420 n(%) |
| --- | --- | --- | --- | --- |
|  | | | | |
| Any Treatment-Emergent AE [1] | 35 ( 35.4) | 75 ( 47.2) | 64 ( 39.5) | 174 ( 41.4) |
|  | | | | |
| Infections and infestations | 20 ( 20.2) | 41 ( 25.8) | 28 ( 17.3) | 89 ( 21.2) |
| COVID-19 | 8 ( 8.1) | 14 ( 8.8) | 13 ( 8.0) | 35 ( 8.3) |
| Nasopharyngitis | 6 ( 6.1) | 14 ( 8.8) | 5 ( 3.1) | 25 ( 6.0) |
| Influenza | 2 ( 2.0) | 4 ( 2.5) | 2 ( 1.2) | 8 ( 1.9) |
| Hordeolum | 1 ( 1.0) | 2 ( 1.3) | 2 ( 1.2) | 5 ( 1.2) |
| Conjunctivitis | 0 | 1 ( 0.6) | 3 ( 1.9) | 4 ( 1.0) |
| Pharyngitis streptococcal | 2 ( 2.0) | 1 ( 0.6) | 1 ( 0.6) | 4 ( 1.0) |
| Sinusitis | 1 ( 1.0) | 3 ( 1.9) | 0 | 4 ( 1.0) |
| Upper respiratory tract infection | 1 ( 1.0) | 2 ( 1.3) | 1 ( 0.6) | 4 ( 1.0) |
| Ear infection | 1 ( 1.0) | 0 | 2 ( 1.2) | 3 ( 0.7) |
| Gastroenteritis viral | 1 ( 1.0) | 1 ( 0.6) | 1 ( 0.6) | 3 ( 0.7) |
| Tonsillitis | 1 ( 1.0) | 0 | 2 ( 1.2) | 3 ( 0.7) |
| Norovirus infection | 0 | 2 ( 1.3) | 0 | 2 ( 0.5) |
| Appendicitis | 0 | 1 ( 0.6) | 0 | 1 ( 0.2) |
| Conjunctivitis bacterial | 1 ( 1.0) | 0 | 0 | 1 ( 0.2) |
| Eye infection | 0 | 1 ( 0.6) | 0 | 1 ( 0.2) |
| Gastroenteritis | 0 | 1 ( 0.6) | 0 | 1 ( 0.2) |
| Gastroenteritis norovirus | 1 ( 1.0) | 0 | 0 | 1 ( 0.2) |
| Helicobacter infection | 0 | 0 | 1 ( 0.6) | 1 ( 0.2) |
| Infection | 0 | 1 ( 0.6) | 0 | 1 ( 0.2) |
| Lower respiratory tract infection | 0 | 1 ( 0.6) | 0 | 1 ( 0.2) |
| Lyme disease | 1 ( 1.0) | 0 | 0 | 1 ( 0.2) |
| Pneumonia | 0 | 0 | 1 ( 0.6) | 1 ( 0.2) |
| Respiratory tract infection | 0 | 1 ( 0.6) | 0 | 1 ( 0.2) |
| Skin infection | 0 | 1 ( 0.6) | 0 | 1 ( 0.2) |
| Varicella | 0 | 0 | 1 ( 0.6) | 1 ( 0.2) |
| Viral infection | 0 | 1 ( 0.6) | 0 | 1 ( 0.2) |
| Viral tonsillitis | 0 | 1 ( 0.6) | 0 | 1 ( 0.2) |
|  |  |  |  |  |
| Injury, poisoning and procedural complications | 4 ( 4.0) | 10 ( 6.3) | 9 ( 5.6) | 23 ( 5.5) |
| Ligament sprain | 1 ( 1.0) | 1 ( 0.6) | 1 ( 0.6) | 3 ( 0.7) |
| Upper limb fracture | 0 | 2 ( 1.3) | 1 ( 0.6) | 3 ( 0.7) |
| Vaccination complication | 0 | 2 ( 1.3) | 1 ( 0.6) | 3 ( 0.7) |
| Foot fracture | 0 | 1 ( 0.6) | 1 ( 0.6) | 2 ( 0.5) |
| Animal scratch | 1 ( 1.0) | 0 | 0 | 1 ( 0.2) |
| Ankle fracture | 0 | 0 | 1 ( 0.6) | 1 ( 0.2) |
| Arthropod bite | 1 ( 1.0) | 0 | 0 | 1 ( 0.2) |
| Corneal abrasion | 0 | 1 ( 0.6) | 0 | 1 ( 0.2) |
| Eye injury | 1 ( 1.0) | 0 | 0 | 1 ( 0.2) |
| Forearm fracture | 0 | 1 ( 0.6) | 0 | 1 ( 0.2) |
| Hand fracture | 0 | 0 | 1 ( 0.6) | 1 ( 0.2) |
| Head injury | 1 ( 1.0) | 0 | 0 | 1 ( 0.2) |
| Intentional overdose | 0 | 0 | 1 ( 0.6) | 1 ( 0.2) |
| Lip injury | 0 | 0 | 1 ( 0.6) | 1 ( 0.2) |
| Muscle strain | 0 | 1 ( 0.6) | 0 | 1 ( 0.2) |
| Procedural pain | 0 | 1 ( 0.6) | 0 | 1 ( 0.2) |
| Torus fracture | 0 | 0 | 1 ( 0.6) | 1 ( 0.2) |
| Ulna fracture | 0 | 0 | 1 ( 0.6) | 1 ( 0.2) |
|  |  |  |  |  |
| Eye disorders | 2 ( 2.0) | 12 ( 7.5) | 8 ( 4.9) | 22 ( 5.2) |
| Dry eye | 0 | 2 ( 1.3) | 3 ( 1.9) | 5 ( 1.2) |
| Retinal degeneration (verbatim term: lattice degeneration) | 0 | 3 ( 1.9) | 1 ( 0.6) | 4 ( 1.0) |
| Conjunctivitis allergic | 0 | 3 ( 1.9) | 0 | 3 ( 0.7) |
| Blepharitis | 0 | 2 ( 1.3) | 0 | 2 ( 0.5) |
| Chalazion | 1 ( 1.0) | 0 | 0 | 1 ( 0.2) |
| Conjunctival hyperaemia | 0 | 0 | 1 ( 0.6) | 1 ( 0.2) |
| Eye irritation | 0 | 0 | 1 ( 0.6) | 1 ( 0.2) |
| Iridocyclitis | 0 | 1 ( 0.6) | 0 | 1 ( 0.2) |
| Noninfective conjunctivitis | 0 | 1 ( 0.6) | 0 | 1 ( 0.2) |
| Papilloedema | 0 | 0 | 1 ( 0.6) | 1 ( 0.2) |
| Punctate keratitis | 0 | 0 | 1 ( 0.6) | 1 ( 0.2) |
| Swelling of eyelid | 1 ( 1.0) | 0 | 0 | 1 ( 0.2) |
| Vision blurred | 0 | 0 | 1 ( 0.6) | 1 ( 0.2) |
|  |  |  |  |  |
| Immune system disorders | 5 ( 5.1) | 8 ( 5.0) | 6 ( 3.7) | 19 ( 4.5) |
| Seasonal allergy | 1 ( 1.0) | 5 ( 3.1) | 4 ( 2.5) | 10 ( 2.4) |
| Immunisation reaction | 3 ( 3.0) | 2 ( 1.3) | 1 ( 0.6) | 6 ( 1.4) |
| Hypersensitivity | 1 ( 1.0) | 1 ( 0.6) | 1 ( 0.6) | 3 ( 0.7) |
|  |  |  |  |  |
| Gastrointestinal disorders | 3 ( 3.0) | 4 ( 2.5) | 6 ( 3.7) | 13 ( 3.1) |
| Vomiting | 1 ( 1.0) | 1 ( 0.6) | 2 ( 1.2) | 4 ( 1.0) |
| Abdominal pain upper | 0 | 1 ( 0.6) | 1 ( 0.6) | 2 ( 0.5) |
| Dental caries | 0 | 0 | 2 ( 1.2) | 2 ( 0.5) |
| Abdominal pain | 0 | 1 ( 0.6) | 0 | 1 ( 0.2) |
| Coeliac disease | 1 ( 1.0) | 0 | 0 | 1 ( 0.2) |
| Constipation | 0 | 1 ( 0.6) | 0 | 1 ( 0.2) |
| Diarrhoea | 0 | 0 | 1 ( 0.6) | 1 ( 0.2) |
| Dyspepsia | 1 ( 1.0) | 0 | 0 | 1 ( 0.2) |
| Food poisoning | 0 | 1 ( 0.6) | 0 | 1 ( 0.2) |
|  |  |  |  |  |
| Respiratory, thoracic and mediastinal disorders | 3 ( 3.0) | 6 ( 3.8) | 4 ( 2.5) | 13 ( 3.1) |
| Oropharyngeal pain | 0 | 2 ( 1.3) | 3 ( 1.9) | 5 ( 1.2) |
| Cough | 0 | 2 ( 1.3) | 2 ( 1.2) | 4 ( 1.0) |
| Asthma | 1 ( 1.0) | 1 ( 0.6) | 0 | 2 ( 0.5) |
| Dyspnoea | 0 | 0 | 1 ( 0.6) | 1 ( 0.2) |
| Rhinorrhoea | 1 ( 1.0) | 0 | 0 | 1 ( 0.2) |
| Sinus congestion | 0 | 1 ( 0.6) | 0 | 1 ( 0.2) |
| Tonsillar hypertrophy | 1 ( 1.0) | 0 | 0 | 1 ( 0.2) |
|  |  |  |  |  |
| General disorders and administration site conditions | 6 ( 6.1) | 4 ( 2.5) | 2 ( 1.2) | 12 ( 2.9) |
| Pyrexia | 2 ( 2.0) | 3 ( 1.9) | 2 ( 1.2) | 7 ( 1.7) |
| Chest pain | 1 ( 1.0) | 0 | 0 | 1 ( 0.2) |
| Complication associated with device | 1 ( 1.0) | 0 | 0 | 1 ( 0.2) |
| Cyst | 0 | 1 ( 0.6) | 0 | 1 ( 0.2) |
| Influenza like illness | 1 ( 1.0) | 0 | 0 | 1 ( 0.2) |
| Injection site pain | 1 ( 1.0) | 0 | 0 | 1 ( 0.2) |
|  |  |  |  |  |
| Nervous system disorders | 1 ( 1.0) | 8 ( 5.0) | 3 ( 1.9) | 12 ( 2.9) |
| Headache | 1 ( 1.0) | 5 ( 3.1) | 2 ( 1.2) | 8 ( 1.9) |
| Migraine | 0 | 1 ( 0.6) | 1 ( 0.6) | 2 ( 0.5) |
| Disturbance in attention | 0 | 1 ( 0.6) | 0 | 1 ( 0.2) |
| Dysarthria | 0 | 1 ( 0.6) | 0 | 1 ( 0.2) |
| Paraesthesia | 0 | 1 ( 0.6) | 0 | 1 ( 0.2) |
| Psychogenic seizure | 0 | 1 ( 0.6) | 0 | 1 ( 0.2) |
| Seizure | 0 | 1 ( 0.6) | 0 | 1 ( 0.2) |
| Syncope | 0 | 1 ( 0.6) | 0 | 1 ( 0.2) |
|  |  |  |  |  |
| Psychiatric disorders | 3 ( 3.0) | 2 ( 1.3) | 7 ( 4.3) | 12 ( 2.9) |
| Anxiety | 0 | 1 ( 0.6) | 2 ( 1.2) | 3 ( 0.7) |
| Attention deficit hyperactivity disorder | 0 | 0 | 3 ( 1.9) | 3 ( 0.7) |
| Depression | 2 ( 2.0) | 0 | 0 | 2 ( 0.5) |
| Childhood depression | 1 ( 1.0) | 0 | 0 | 1 ( 0.2) |
| Disruptive mood dysregulation disorder | 1 ( 1.0) | 0 | 0 | 1 ( 0.2) |
| Intentional self-injury | 1 ( 1.0) | 0 | 0 | 1 ( 0.2) |
| Mania | 0 | 0 | 1 ( 0.6) | 1 ( 0.2) |
| Nightmare | 1 ( 1.0) | 0 | 0 | 1 ( 0.2) |
| Oppositional defiant disorder | 1 ( 1.0) | 0 | 0 | 1 ( 0.2) |
| Panic attack | 0 | 0 | 1 ( 0.6) | 1 ( 0.2) |
| Sleep disorder | 0 | 0 | 1 ( 0.6) | 1 ( 0.2) |
| Suicide attempt | 0 | 1 ( 0.6) | 0 | 1 ( 0.2) |
|  |  |  |  |  |
| Skin and subcutaneous tissue disorders | 2 ( 2.0) | 2 ( 1.3) | 4 ( 2.5) | 8 ( 1.9) |
| Acne | 1 ( 1.0) | 2 ( 1.3) | 3 ( 1.9) | 6 ( 1.4) |
| Dermatitis | 1 ( 1.0) | 0 | 1 ( 0.6) | 2 ( 0.5) |
|  |  |  |  |  |
| Metabolism and nutrition disorders | 2 ( 2.0) | 2 ( 1.3) | 2 ( 1.2) | 6 ( 1.4) |
| Iron deficiency | 2 ( 2.0) | 0 | 2 ( 1.2) | 4 ( 1.0) |
| Glucose tolerance impaired | 0 | 1 ( 0.6) | 0 | 1 ( 0.2) |
| Vitamin D deficiency | 0 | 1 ( 0.6) | 0 | 1 ( 0.2) |
|  |  |  |  |  |
| Musculoskeletal and connective tissue disorders | 1 ( 1.0) | 1 ( 0.6) | 3 ( 1.9) | 5 ( 1.2) |
| Arthralgia | 0 | 0 | 2 ( 1.2) | 2 ( 0.5) |
| Growth retardation | 0 | 0 | 1 ( 0.6) | 1 ( 0.2) |
| Musculoskeletal chest pain | 0 | 0 | 1 ( 0.6) | 1 ( 0.2) |
| Pain in extremity | 0 | 1 ( 0.6) | 0 | 1 ( 0.2) |
| Tendonitis | 1 ( 1.0) | 0 | 0 | 1 ( 0.2) |
|  |  |  |  |  |
| Reproductive system and breast disorders | 1 ( 1.0) | 2 ( 1.3) | 0 | 3 ( 0.7) |
| Dysmenorrhoea | 0 | 2 ( 1.3) | 0 | 2 ( 0.5) |
| Heavy menstrual bleeding | 1 ( 1.0) | 0 | 0 | 1 ( 0.2) |
|  |  |  |  |  |
| Investigations | 0 | 1 ( 0.6) | 1 ( 0.6) | 2 ( 0.5) |
| Intraocular pressure increased | 0 | 0 | 1 ( 0.6) | 1 ( 0.2) |
| SARS-CoV-2 test positive | 0 | 1 ( 0.6) | 0 | 1 ( 0.2) |
|  |  |  |  |  |
| Blood and lymphatic system disorders | 1 ( 1.0) | 0 | 0 | 1 ( 0.2) |
| Anaemia | 1 ( 1.0) | 0 | 0 | 1 ( 0.2) |
|  |  |  |  |  |
| Congenital, familial and genetic disorders | 0 | 1 ( 0.6) | 0 | 1 ( 0.2) |
| Supernumerary teeth | 0 | 1 ( 0.6) | 0 | 1 ( 0.2) |
|  |  |  |  |  |
| Ear and labyrinth disorders | 1 ( 1.0) | 0 | 0 | 1 ( 0.2) |
| Ear congestion | 1 ( 1.0) | 0 | 0 | 1 ( 0.2) |
|  |  |  |  |  |
| Neoplasms benign, malignant and unspecified (incl cysts and polyps) | 1 ( 1.0) | 0 | 0 | 1 ( 0.2) |
| Skin papilloma | 1 ( 1.0) | 0 | 0 | 1 ( 0.2) |

**Supplemental Table 4: Normalised SER or AL change from Stage 2 baseline at Month 48 (Stage 2 mITT set).**

| Descriptive  Statistics | Atropine Sulphate 0.01% to 0.01% N=29 | Atropine Sulphate 0.01% to Vehicle N=31 | Atropine Sulphate 0.01% to 0.02% N=33 | Atropine Sulphate 0.02% to 0.02% N=56 | Atropine Sulphate 0.02% to Vehicle N=52 | Atropine Sulphate 0.02% to 0.01% N=52 | Vehicle to Atropine Sulphate 0.01%  N=54 | Vehicle to Atropine Sulphate 0.02%  N=54 |
| --- | --- | --- | --- | --- | --- | --- | --- | --- |
| SER change from Stage 2 Baseline, combined left and right eyes | | | | | | | | |
| n (eyes) | 54 | 52 | 58 | 102 | 96 | 90 | 94 | 98 |
| Median, D | -0.17 | -0.21 | -0.18 | -0.16 | -0.19 | -0.18 | -0.16 | -0.16 |
| Min, Max | -0.76, 0.24 | -0.75, 0.78 | -0.84, 0.92 | -1.31, 0.66 | -0.94, 0.94 | -1.66, 0.69 | -1.00, 0.80 | -1.86, 1.64 |
| AL change from Stage 2 Baseline, combined left and right eyes | | | | | | | | |
| n (eyes) | 57 | 62 | 62 | 106 | 97 | 100 | 104 | 108 |
| Median, mm | 0.10 | 0.11 | 0.07 | 0.14 | 0.13 | 0.11 | 0.11 | 0.10 |
| Min, Max | -0.12, 0.54 | -0.06, 0.45 | -0.13. 0.33 | -0.08, 0.55 | -0.20, 0.69 | -0.12, 1.12 | -0.10, 0.59 | -0.06, 0.76 |

AL, axial length. D, dioptre. Max, maximum. Min, minimum. mITT, modified intent-to-treat. SER, spherical equivalent refractive error.

**Supplemental Table 5: Normalised SER or AL change from Stage 2 baseline at Month 48 (Stage 2 ITT set).**

| Descriptive  Statistics | Atropine Sulphate 0.01% to 0.01% N=38 | Atropine Sulphate 0.01% to Vehicle N=39 | Atropine Sulphate 0.01% to 0.02% N=39 | Atropine Sulphate 0.02% to 0.02% N=63 | Atropine Sulphate 0.02% to Vehicle N=60 | Atropine Sulphate 0.02% to 0.01% N=59 | Vehicle to Atropine Sulphate 0.01%  N=62 | Vehicle to Atropine Sulphate 0.02%  N=60 |
| --- | --- | --- | --- | --- | --- | --- | --- | --- |
| SER change from Stage 2 baseline, combined left and right eyes | | | | | | | | |
| n (eyes) | 70 | 68 | 66 | 116 | 108 | 100 | 108 | 106 |
| Median, D | -0.15 | -0.23 | -0.15 | -0.15 | -0.18 | -0.19 | -0.15 | -0.17 |
| Min, Max | -0.76, 1.39 | -1.43, 0.78 | -0.84, 0.92 | -1.31, 0.66 | -0.94, 0.94 | -1.66, 0.79 | -1.00, 0.80 | -1.86, 1.64 |
| AL change from Stage 2 baseline, combined left and right eyes | | | | | | | | |
| n (eyes) | 75 | 78 | 72 | 120 | 113 | 114 | 120 | 116 |
| Median, mm | 0.08 | 0.11 | 0.06 | 0.13 | 0.13 | 0.11 | 0.11 | 0.11 |
| Min, Max | -0.12, 0.54 | -0.06, 0.51 | -0.21, 0.33 | -0.08, 0.55 | -0.20, 1.16 | -0.12, 1.12 | -0.10, 0.59 | -0.06, 0.75 |

AL, axial length. D, dioptre. ITT, intent-to-treat. Max, maximum. Min, minimum. SER, spherical equivalent refractive error.

Supplemental Table 6: Proportion of Participants that Progressed to High Myopia (Normalised SER ≤ ‑6.00 D) by Month 48 (Stage 2 mITT set)

| Statistic | Vehicle to Atropine Sulphate 0.01% N=54 n (%) | Vehicle to Atropine Sulphate 0.02% N=54 n (%) | Atropine Sulphate 0.01% to Vehicle N=31 n (%) | Atropine Sulphate 0.01% to 0.01% N=29 n (%) | Atropine Sulphate 0.01% to 0.02% N=33 n (%) | Atropine Sulphate 0.02% to 0.01% N=52 n (%) | Atropine Sulphate 0.02% to Vehicle N=52 n (%) | Atropine Sulphate 0.02% to 0.02% N=56 n (%) |
| --- | --- | --- | --- | --- | --- | --- | --- | --- |
| Progressed to High Myopiaa | | | | | | | | |
| No | 42 (87.5) | 46 (93.9) | 25 (96.2) | 24 (88.9) | 24 (82.8) | 40 (87.0) | 40 (83.3) | 47 (92.2) |
| Yes | 6 (12.5) | 3 (6.1) | 1 (3.8) | 3 (11.1) | 5 (17.2) | 6 (13.0) | 8 (16.7) | 4 (7.8) |
| Absolute risk difference (continuing vs switched to vehicle) | -- | -- | 7.3% | | -- | -- | -8.9% | |
| 95% Wald CI | -- | -- | -7%, 21% | | -- | -- | -22%, 4% | |
| p value (continuing vs switched to vehicle) | -- | -- | 0.3 | | -- | -- | 0.18 | |

CI, confidence interval; D=dioptre; mITT=Modified Intent-to-Treat; N=number of participants in population; n=number of participants per category of progression; SER=spherical equivalent refraction

^a^ Participants who progressed to high myopia in either or both eyes.

**Supplemental Table 7: Summary of proportion of participants’ eyes that show less than 0.75 D myopia progression from Stage 2 baseline (Stage 2 mITT set).**

|  | Atropine Sulphate 0.01% to Vehicle N=31 n (%) | Atropine Sulphate 0.02% to Vehicle N=52 n (%) | Vehicle to Atropine Sulphate 0.01% N=54 n (%) | Vehicle to Atropine Sulphate 0.02% N=54 n (%) | Atropine Sulphate 0.01% to 0.01% N=29 n (%) | Atropine Sulphate 0.01% to 0.02% N=33 n (%) | Atropine Sulphate 0.02% to 0.01% N=52 n (%) | Atropine Sulphate 0.02% to 0.02% N=56 n (%) |
| --- | --- | --- | --- | --- | --- | --- | --- | --- |
| Less than 0.75 D myopia progression at Month 48, n (%) | | | | | | | | |
| Yes | 49 (98.0) | 91 (94.8) | 88 (93.6) | 95 (96.9) | 52 (96.3) | 56 (96.6) | 81 (90.0) | 92 (92.0) |
| No | 1 (2.0) | 5 (5.2) | 6 (6.4) | 3 (3.1) | 2 (3.7) | 2 (3.4) | 9 (10.0) | 8 (8.0) |

D=dioptre; mITT=Modified Intent-to-Treat.

**Supplemental Table 8: Summary of crystalline lens thickness measurements at Stage 2 baseline and Month 48 (Stage 2 ITT set).**

| Descriptive  Statistics | Atropine Sulphate 0.01% to 0.01% N=38 | Atropine Sulphate 0.01% to Vehicle N=39 | Atropine Sulphate 0.01% to 0.02% N=39 | Atropine Sulphate 0.02% to 0.02% N=63 | Atropine Sulphate 0.02% to Vehicle N=60 | Atropine Sulphate 0.02% to 0.01% N=59 | | Vehicle to Atropine Sulphate 0.01%  N=62 | Vehicle to Atropine Sulphate 0.02%  N=60 |
| --- | --- | --- | --- | --- | --- | --- | --- | --- | --- |
| Stage 2 baseline, combined left and right eyes | | | | | | | | | |
| n (eyes) | 44 | 49 | 52 | 92 | 82 | | 64 | 70 | 72 |
| Median, mm | 3.40 | 3.37 | 3.33 | 3.31 | 3.37 | | 3.34 | 3.40 | 3.37 |
| Min, Max | 2.90, 3.71 | 2.61, 3.75 | 2.98, 3.67 | 1.42, 3.77 | 2.74, 3.83 | | 2.79, 3.90 | 3.10, 3.80 | 2.69, 3.76 |
| Month 48, combined left and right eyes | | | | | | | | | |
| n (eyes) | 38 | 48 | 42 | 74 | 74 | | 55 | 67 | 59 |
| Median, mm | 3.41 | 3.45 | 3.39 | 3.32 | 3.40 | | 3.35 | 3.40 | 3.38 |
| Min, Max | 2.90, 3.75 | 2.99, 3.71 | 3.00, 3.62 | 2.97, 3.81 | 3.03, 3.82 | | 2.68, 3.63 | 3.09, 3.73 | 2.69, 3.71 |
| Month 48 change from Stage 2 baseline, combined left and right eyes | | | | | | | | | |
| n (eyes) | 38 | 46 | 42 | 72 | 74 | | 54 | 65 | 59 |
| Median, mm | 0.030 | 0.020 | 0.010 | 0.010 | 0.020 | | 0.020 | 0.010 | 0.010 |
| Min, Max | -0.16, 0.42 | -0.17, 1.08 | -0.31, 0.18 | -0.25, 0.21 | -0.22, 0.74 | | -0.65, 0.09 | -0.09, 0.18 | -0.69, 3.42 |

ITT, intent-to-treat. Min, minimum. Max, maximum.

**Supplemental Table 9: Summary of pupil size measurements at Stage 2 baseline and Month 48 (Stage 2 ITT set).**

| Descriptive  Statistics | Atropine Sulphate 0.01% to 0.01% N=38 | Atropine Sulphate 0.01% to Vehicle N=39 | Atropine Sulphate 0.01% to 0.02% N=39 | Atropine Sulphate 0.02% to 0.02% N=63 | Atropine Sulphate 0.02% to Vehicle N=60 | Atropine Sulphate 0.02% to 0.01% N=59 | | Vehicle to Atropine Sulphate 0.01%  N=62 | Vehicle to Atropine Sulphate 0.02%  N=60 |
| --- | --- | --- | --- | --- | --- | --- | --- | --- | --- |
| Stage 2 baseline, right eye | | | | | | | | | |
| n (eyes) | 38 | 39 | 39 | 63 | 60 | | 58 | 61 | 60 |
| Mean, mm | 4.89 | 4.91 | 4.85 | 4.96 | 5.03 | | 4.92 | 4.66 | 4.55 |
| SD | 1.16 | 0.90 | 0.93 | 1.03 | 0.97 | | 1.15 | 0.96 | 0.93 |
| Month 48, right eye | | | | | | | | | |
| n (eyes) | 38 | 39 | 36 | 60 | 57 | | 58 | 61 | 58 |
| Mean, mm | 4.57 | 4.42 | 5.07 | 4.76 | 4.45 | | 4.59 | 4.89 | 4.81 |
| SD | 1.08 | 0.89 | 0.88 | 1.14 | 0.99 | | 1.15 | 1.00 | 0.94 |
| Month 48 change from Stage 2 baseline, right eye | | | | | | | | | |
| n (eyes) | 38 | 39 | 36 | 60 | 57 | | 57 | 60 | 58 |
| Mean, mm | -0.32 | -0.49 | 0.20 | -0.21 | -0.54 | | -0.31 | 0.22 | 0.26 |
| SD | 0.87 | 0.69 | 0.72 | 0.73 | 0.75 | | 0.83 | 0.69 | 0.68 |
| Stage 2 baseline, left eye | | | | | | | | | |
| n (eyes) | 38 | 39 | 39 | 63 | 60 | | 58 | 61 | 60 |
| Mean, mm | 4.89 | 4.90 | 4.83 | 4.96 | 5.08 | | 4.92 | 4.69 | 4.54 |
| SD | 1.15 | 0.95 | 0.92 | 1.05 | 1.05 | | 1.16 | 0.97 | 0.91 |
| Month 48, left eye | | | | | | | | | |
| n (eyes) | 38 | 39 | 36 | 60 | 57 | | 58 | 61 | 58 |
| Mean, mm | 4.61 | 4.44 | 5.01 | 4.73 | 4.45 | | 4.63 | 4.90 | 4.79 |
| SD | 1.12 | 0.93 | 0.81 | 1.13 | 0.97 | | 1.18 | 1.02 | 0.94 |
| Month 48 change from Stage 2 baseline, left eye | | | | | | | | | |
| n (eyes) | 38 | 39 | 36 | 60 | 57 | | 57 | 60 | 58 |
| Mean, mm | -0.29 | -0.46 | 0.15 | -0.24 | -0.57 | | -0.27 | 0.20 | 0.25 |
| SD | 0.79 | 0.70 | 0.57 | 0.72 | 0.77 | | 0.77 | 0.68 | 0.65 |

ITT, intent-to-treat.
